# Hypericin Photodynamic Therapy Induces Cytotoxicity and Modulates Cytokine Secretion in MCF-7 Breast Cancer Cells

**DOI:** 10.3390/jcm14217514

**Published:** 2025-10-23

**Authors:** Magdalena Czarnecka-Czapczyńska, Zenon Czuba, David Aebisher, Wiktoria Mytych, Jakub Fiegler-Rudol, Rafał Wiench, Aleksandra Kawczyk-Krupka

**Affiliations:** 1 Doctoral School, Medical University of Silesia, 40-055 Katowice, Poland; magdalena.czarnecka921114@gmail.com; 2 Department of Internal Diseases, Angiology and Physical Medicine, Center for Laser Diagnostics and Therapy, Faculty of Medical Sciences in Zabrze, Medical University of Silesia, 40-055 Katowice, Poland; 3 Department of Microbiology and Immunology, Faculty of Medical Sciences in Zabrze, Medical University of Silesia, 40-055 Katowice, Poland; zczuba@sum.edu.pl; 4 Department of Photomedicine and Physical Chemistry, Medical College, Rzeszów University, 35-310 Rzeszów, Poland; 5 English Division Science Club, Medical College, Rzeszów University, 35-310 Rzeszów, Poland; wiktoriamytych@gmail.com; 6 Department of Periodontal and Oral Mucosa Diseases, Faculty of Medical Sciences in Zabrze, Medical University of Silesia, 40-055 Katowice, Poland; s88998@365.sum.edu.pl (J.F.-R.); rwiench@sum.edu.pl (R.W.)

**Keywords:** photodynamic therapy, hypericin, breast cancer, MCF-7, cytokines, IL-6, IL-8, TNF-α

## Abstract

**Background/Aim**: Photodynamic therapy uses a photosensitizer and light to generate reactive oxygen species that kill tumor cells and can shift inflammatory signaling. Hypericin is a potent photosensitizer, but its immunomodulatory impact in breast cancer needs clarification. We evaluated the phototoxic and cytokine-modulating effects of hypericin-mediated photodynamic therapy in MCF-7 human breast adenocarcinoma cells. This study examines how HYP-PDT affects MCF-7 breast cancer cells by assessing viability and cytokine secretion to guide the development of targeted, immune-enhancing PDT protocols. **Methods**: MCF-7 cells were incubated with hypericin at 0, 0.125, 0.25, 0.5, or 1 μM, then exposed to light doses of 0, 1, 2, or 5 J/cm^2^. Viability was measured 24 h later by MTT; selected conditions were also assessed by Trypan Blue. Cell supernatants collected after sublethal treatment were analyzed for IL-6, IL-8, IL-10, and TNF-α using a multiplex immunoassay. Experiments were repeated four times. Statistical analyses followed the study’s plan for group comparisons. **Results**: At 1 J/cm^2^, MTT values did not differ from matched dark controls across hypericin concentrations. At 2 and 5 J/cm^2^, some conditions showed increased MTT signal relative to controls, indicating higher metabolic activity; Trypan Blue performed at 0 J/cm^2^ showed a concentration-dependent reduction in viability with hypericin. Hypericin-PDT decreased IL-6 and IL-8 concentrations and increased TNF-α in MCF-7 supernatants. No statistically significant changes were detected for IL-10. **Conclusions**: Hypericin-PDT altered inflammatory readouts in MCF-7 cells, with reductions in IL-6 and IL-8 and an increase in TNF-α, consistent with a pro-inflammatory shift. Viability results suggest condition-dependent changes in metabolic activity or survival effects that warrant confirmation with matched cell counts across all light doses. These findings support further standardized dosimetry and multi-line validation of hypericin-PDT in breast cancer models.

## 1. Introduction

### 1.1. Rationale

Breast cancer (BC) remains one of the most prevalent and life-threatening malignancies among women worldwide, representing a major public health challenge. Patients diagnosed with BC often require multimodal treatment strategies that include surgery, chemotherapy, hormone therapy, biological agents, and radiation therapy [1]. These treatments, while effective, are frequently associated with severe side effects that negatively impact quality of life. Therefore, there is an ongoing need for therapeutic approaches that are not only effective but also minimally invasive and associated with fewer adverse effects [2]. In recent years, the tumor microenvironment and immune signaling have been recognized as key factors in BC development and progression. Cytokines play a pivotal role in modulating tumor growth, angiogenesis, and immune evasion. Various cytokines, such as IL-1, IL-2, IL-6, IL-8, IL-10, IL-11, IL-12, IL-17A, IL-18, IL-19, IL-20, IL-23, IRF-1, IRF-2, IFN-γ, TGF-β, and gp130, have been implicated in BC pathogenesis [3,4,5]. Among these, IL-2, IL-6, IL-8, and IL-10 are of particular interest due to their complex roles in tumor-promoting inflammation and immune regulation, making them potential therapeutic targets [3,4,5]. Photodynamic therapy (PDT) has emerged as a promising minimally invasive treatment for various cancers, including BC. PDT involves three essential components: a photosensitizer (PS), light of an appropriate wavelength, and oxygen. Upon light activation, the PS generates singlet oxygen (^1^O_2_), a highly reactive form of oxygen that selectively induces tumor cell death through apoptosis or necrosis [6]. Compared to conventional surgical treatments, PDT is less invasive and can be repeated multiple times, making it an attractive therapeutic option for localized tumors [7]. The choice of PS is a critical determinant of PDT efficacy. Ideal PSs should exhibit strong phototoxicity, minimal dark toxicity, availability, and sufficient tissue penetration [8,9]. Clinically approved PSs include Photofrin, Levulan, Metvix, Foscan, Laserphyrin, and Visudyne [10,11,12], while newer candidates such as HPPH, SnEt2, and LuTex are undergoing preclinical and clinical evaluation [13]. Hypericin (HYP), a naturally occurring hydrophobic compound, is a potent PS with established anticancer activity. It has demonstrated promising effects in treating various cancers, including breast, colon, bladder, cervical, glioma, leukemia, melanoma, and lymphoma [14,15,16]. Upon activation, HYP participates in both Type I and Type II photochemical reactions. Type I PDT involves electron or proton transfer, generating reactive oxygen species (ROS) such as hydrogen peroxide (H_2_O_2_), hydroxyl radicals (OH·), and superoxide anions (O_2_·^−^). Type II PDT, the predominant mechanism for HY, involves direct energy transfer to molecular oxygen, producing singlet oxygen (^1^O_2_), which damages tumor cell membranes, mitochondria, and DNA [17]. These processes ultimately lead to irreversible tumor cell destruction. The effectiveness of PDT depends not only on the PS and light dose but also on tissue optical properties. Light in the near-infrared range (700–1100 nm) penetrates more deeply into tissues due to lower absorption by endogenous chromophores such as oxyhemoglobin, deoxyhemoglobin, melanin, and lipids [18]. Optimizing light delivery is therefore essential for treating tumors located beneath the skin surface. Beyond direct tumor cytotoxicity, PDT has been increasingly recognized for its capacity to modulate the immune system. Tumors often create an immunosuppressive microenvironment that hinders antitumor immunity [19]. PDT can disrupt this immunosuppressive milieu by inducing immunogenic cell death and releasing danger-associated molecular patterns (DAMPs), leading to cytokine production and activation of immune cells. Our previous research demonstrated that HYP-PDT effectively reduced cytokine levels such as IL-8, IL-11, IL-19, IL-22, and metalloproteinase-1 (MMP-1) in skin cancer cell cultures, indicating an immunomodulatory effect in vitro [20]. However, to date, there has been no comprehensive evaluation of HYP-PDT on breast cancer cell lines, particularly regarding its effect on cytokine secretion.

### 1.2. Objectives

This research addresses a critical gap by investigating the dual phototoxic and immunomodulatory effects of HYP-PDT in MCF-7 human breast adenocarcinoma cells, focusing on cytokines IL-6, IL-8, IL-10, and TNF-α. As a preliminary study, this work was conducted using a simplified in vitro monoculture model to generate foundational data before progressing to more complex co-culture systems that include dendritic cells and T cells to better reflect tumor-immune interactions. Understanding how HYP-PDT influences both cancer cell viability and cytokine signaling is essential for determining its therapeutic potential not only as a cytotoxic modality but also as a strategy to stimulate antitumor immunity. By elucidating these mechanisms, this study provides foundational data that may guide future preclinical and clinical applications of HYP-PDT in breast cancer treatment. The aim of this study was to evaluate the effects of HYP-PDT on MCF-7 cells in vitro by assessing changes in cell viability and cytokine secretion profiles. The results obtained may contribute to the development of new, targeted PDT protocols for breast cancer with improved efficacy and reduced side effects.

## 2. Materials and Methods

### 2.1. Study Design

This in vitro experimental study was designed to evaluate the phototoxic and immunomodulatory effects of HYP-PDT on human breast cancer adenocarcinoma cells (MCF-7). The study consisted of four main experimental phases:

1. Cell culture and preparation of the MCF-7 breast cancer cell line.
2. Hypericin treatment at different concentrations (0–1.0 µM).
3. Photodynamic therapy, using defined light doses (0, 1, 2, and 5 J/cm^2^).
4. Outcome measurements, including:
  - Cell viability using the MTT assay and Trypan Blue exclusion test.
  - Quantification of cytokines (IL-6, IL-8, IL-10, TNF-α) in culture supernatants.

Control groups included:

- Untreated cells (no hypericin, no light).
- Cells treated with hypericin but kept in the dark (dark controls).
- Cells exposed to light without hypericin (light-only controls).

Each experimental condition was performed in quadruplicate (*n* = 4 independent experiments).

This study was designed as a preliminary in vitro investigation focused solely on MCF-7 breast cancer cells, with the goal of generating foundational data on the cytotoxic and immunomodulatory effects of HY-PDT before expanding to more complex co-culture systems incorporating immune cells.

### 2.2. Chemicals and Reagents

All chemicals were of analytical grade. Hypericin (HY), dimethyl sulfoxide (DMSO), MTT, and hydrocortisone were purchased from Sigma-Aldrich (St. Louis, MO, USA). Dulbecco’s Modified Eagle’s Medium: F-12 (DMEM/F12), fetal bovine serum (FBS), trypsin, and ethylenediaminetetraacetic acid (EDTA) were obtained from the American Type Culture Collection (ATCC, Manassas, VA, USA). Dulbecco’s phosphate-buffered saline (PBS) without calcium and magnesium was obtained from Sigma-Aldrich (Warsaw, Poland). The Bio-Plex™ Pro Human Cytokine Assay kit was purchased from Bio-Rad Laboratories, Inc. (Hercules, CA, USA). Cell images were captured with an Olympus IX51 microscope equipped with a color view camera (Olympus Inc., Tokyo, Japan) and analyzed using Cell F version 2.6 software (Soft Imaging System GmbH, Münster, Germany). Light energy was measured using a Newport 1918-C power meter (Newport, Franklin, MA, USA). Infrared (IR) filters were purchased from Thorlabs GmbH (Berlin, Germany).

While MTT provided an initial readout of metabolic activity, we recognize its limitations in distinguishing between cell death and altered redox states. Complementary assays such as flow cytometry for apoptosis/necrosis, caspase activity assays, and LDH release are planned to more directly quantify cell death mechanisms.

### 2.3. Cell Line and Culture Conditions

The human breast cancer adenocarcinoma cell line MCF-7 (ATCC^®^ HTB-22™) was purchased from ATCC. This cell line was originally derived from a 69-year-old female patient diagnosed with breast adenocarcinoma.

Cells were cultured in Eagle’s Minimum Essential Medium (EMEM) supplemented with:

- 2 mM L-glutamine
- 1 mM sodium pyruvate
- 1500 mg/L sodium bicarbonate
- 0.01 mg/mL human recombinant insulin
- 10% FBS

Cultures were maintained at 37 °C in a humidified incubator containing 5% CO_2_. The growth medium was replaced every 3 days. For passaging, cells were detached using 0.25% trypsin/0.53 mM EDTA solution.

### 2.4. Hypericin Preparation and Treatment

A 1 mM hypericin stock solution was prepared in DMSO and stored at −20 °C, protected from light. Working solutions of 0.125, 0.25, 0.5, and 1.0 µM were freshly prepared in complete culture medium immediately before use. The final DMSO concentration in all treatments did not exceed 0.1% (*v*/*v*). Cells were seeded at 20,000 cells/well in 96-well plates and allowed to adhere for 24 h. After adherence, the medium was replaced with fresh medium containing hypericin at the specified concentrations and incubated for 2 h in the dark to prevent premature photoactivation. Untreated cells served as negative controls. To confirm hypericin uptake, fluorescence imaging was performed using the Olympus IX51 microscope.

### 2.5. PDT Procedure

PDT was performed using an incoherent TO-1 photodynamic therapy lamp (Cosmedico Medizintechnik GmbH, Schwenningen, Germany) equipped with orange and infrared filters to deliver light in the 580–720 nm wavelength range [20].

- Irradiance at the cell surface: 35 mW/cm^2^ (verified using Newport power meter).
- Light doses applied: 1, 2, and 5 J/cm^2^.
- Distance from light source to cells: 30 cm.

To prevent heat-induced cell damage, a double water filter was placed in the optical path. Control plates were kept covered in the dark during irradiation.

Following irradiation, all plates were incubated for 24 h in the dark before downstream assays. Spectral output at the plate plane was verified with the instrument’s filters to fall within 580 to 720 nm. Irradiance at the well plane was 35 mW/cm^2^ with spatial variation within ±5% across the plate. Medium temperature at the cell layer remained at 37.0 ± 0.5 °C during exposure.

### 2.6. Cell Viability Assessment—MTT Assay

Cell metabolic activity was measured using the MTT assay. After 24 h incubation post-PDT, culture supernatants were carefully collected for cytokine analysis and stored at −80 °C. Fresh medium containing 0.5 mg/mL MTT was added to each well and incubated for 4 h at 37 °C. The resulting purple formazan crystals were dissolved by adding 200 µL DMSO to each well and shaking for 10 min.

- A 150 µL aliquot from each well was transferred to a new 96-well plate.
- Absorbance was read at 550 nm using a microplate reader (ELx800, Bio-Tek Instruments Inc., Winooski, VT, USA).

Cell viability (%) was calculated using the formula:

Viability (%) = 100 × OD_550_e /OD_550_b

where OD_550_e = Mean optical density of treated wells. OD_550_b = Mean optical density of untreated control wells. For cytokine analyses, supernatants were collected from parallel wells not exposed to MTT reagents.

### 2.7. Trypan Blue Exclusion Assay

To confirm the MTT results, a Trypan Blue exclusion assay was performed on selected samples at 0 J/cm^2^ light dose. MCF-7 cells were washed twice with cold PBS and stained with 0.4% (*w*/*v*) Trypan Blue solution. Viable (unstained) and non-viable (stained) cells were counted manually using a hemocytometer under a light microscope. The untreated control was set to 100% viability, and treatment groups were expressed as a percentage relative to the control.

### 2.8. Cytokine Quantification

The collected supernatants were analyzed for IL-6, IL-8, IL-10, and TNF-α concentrations using the Bio-Plex™ Pro Human Cytokine Assay kit (Bio-Rad). Assays were performed following the manufacturer’s instructions, using magnetic bead-based sandwich immunoassays. Fluorescence was measured on the Bio-Plex 3D Suspension Array System, and cytokine concentrations were calculated using standard curves generated in Bio-Plex Manager Software 6.2. Cytokine concentrations were normalized to viable cell number measured in sister wells by Trypan Blue at the time of supernatant collection.

### 2.9. Statistical Analysis

All experiments were repeated independently four times (n = 4). Data are presented as mean ± standard deviation (SD).

- Normality of data was assessed using the Shapiro–Wilk test.
- For MTT results, comparisons between groups and controls were made using Student’s *t*-test.
- Cytokine data were analyzed using Kruskal–Wallis ANOVA, followed by a multiple comparison post hoc test.

Statistical significance was set at *p* < 0.05. Analyses were performed using Statistica version 13 (TIBCO Software Inc., Palo Alto, CA, USA). Graphs were created in Microsoft Excel (version 2303).

### 2.10. Summary of Experimental Workflow

1. Seed MCF-7 cells → 24 h adherence.
2. Incubate with hypericin for 2 h in the dark.
3. Irradiate with the specified light dose or keep as dark/light control.
4. Incubate 24 h post-PDT in dark.
5. Collect supernatants → cytokine assay.
6. Perform MTT and Trypan Blue viability assays.
7. Analyze results statistically.

## 3. Results

### 3.1. Effect of HYP-PDT on MCF-7 Cell Viability

**Figure 1:**
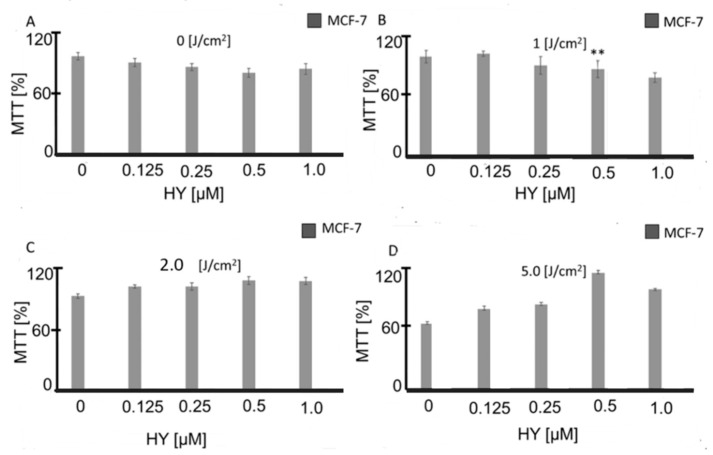
MTT reduction [%] in the MCF-7 cell line for different Hypericin concentrations: 0 μM, 0.125 μM, 0.25 μM, 0.5 μM, and 1 μM, and light dose (**A**) 0 J/cm^2^; (**B**) 1 J/cm^2^; (**C**) 2 J/cm^2^; and (**D**) 5 J/cm^2^. The values represent the means ± SD. (**A**) *p* > 0.05. (**B**) ** *p* < 0.05; (**C**) *p* > 0.05. (**D**) *p* > 0.05. In the study, the reduction of MTT was calculated as a percentage of the dark group control for each cell line in %.

The cytotoxicity of hypericin-mediated photodynamic therapy (HY-PDT) was first assessed by measuring MCF-7 cell viability using the MTT assay. At a light dose of 1 J/cm^2^, no significant differences in viability were observed compared to dark and light-only control groups, regardless of hypericin concentration (0, 0.125, 0.25, 0.5, or 1 µM) (*p* > 0.05). At 2 and 5 J/cm^2^, several conditions showed higher MTT signal relative to controls, consistent with increased mitochondrial reductase activity. This readout does not by itself indicate increased survival or proliferation. This increase suggests a dose-dependent effect of HYP-PDT on cellular metabolic activity (Figure 1). To dissociate metabolic signal from survival, future work should include DNA or protein content assays, direct cell counts, colony formation, and ATP-based viability. MTT can overestimate viability when mitochondrial activity is transiently elevated after oxidative stress. The divergence from Trypan Blue at some doses suggests redox adaptation rather than a growth advantage.

### 3.2. Validation of Viability by Trypan Blue Exclusion

To confirm MTT results, a Trypan Blue exclusion assay was performed at 0 J/cm^2^ light dose. Results showed a concentration-dependent reduction in viable MCF-7 cells when treated with increasing hypericin concentrations.

- At 0.125 µM HY, viability decreased slightly to 95 ± 2.3%,
- At 0.5 µM HY, viability dropped to 84 ± 15%,
- At 1.0 µM HY, viability was 87 ± 5% compared to 98.9 ± 0.3% in untreated controls (Table 1).

**Figure 2:**
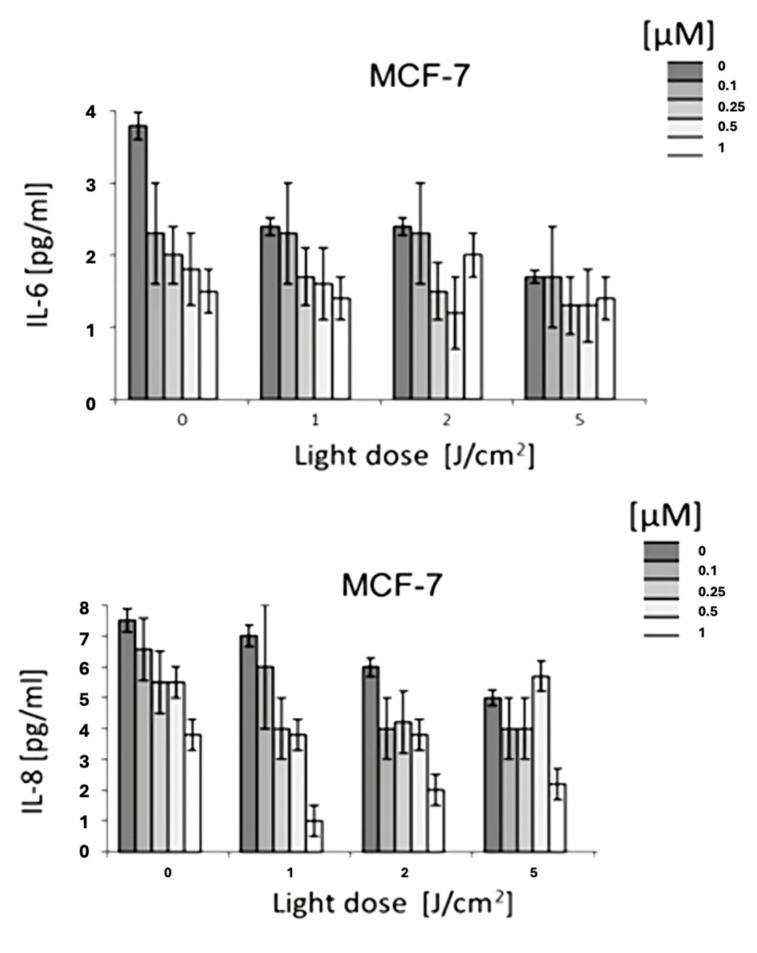
The concentration of IL-6 and IL-8 in the MCF-7 cell line. Both figures are the results of various light doses (0 J/cm^2^, 1 J/cm^2^, 2 J/cm^2^, 5 J/cm^2^) and the results of various Hypericin concentrations (0 μM, 0.125 μM, 0.25 μM, 0.5 μM). The values represent the means ± SD.

The trends observed in the Trypan Blue assay were consistent with MTT findings, confirming that hypericin alone reduced MCF-7 cell survival in a dose-dependent manner (Figure 2).

### 3.3. Cytokine Modulation by HY-PDT

The immunomodulatory effect of HYP-PDT was evaluated by measuring cytokine concentrations (IL-6, IL-8, IL-10, and TNF-α) in culture supernatants collected 24 h after treatment.

**Figure 3:**
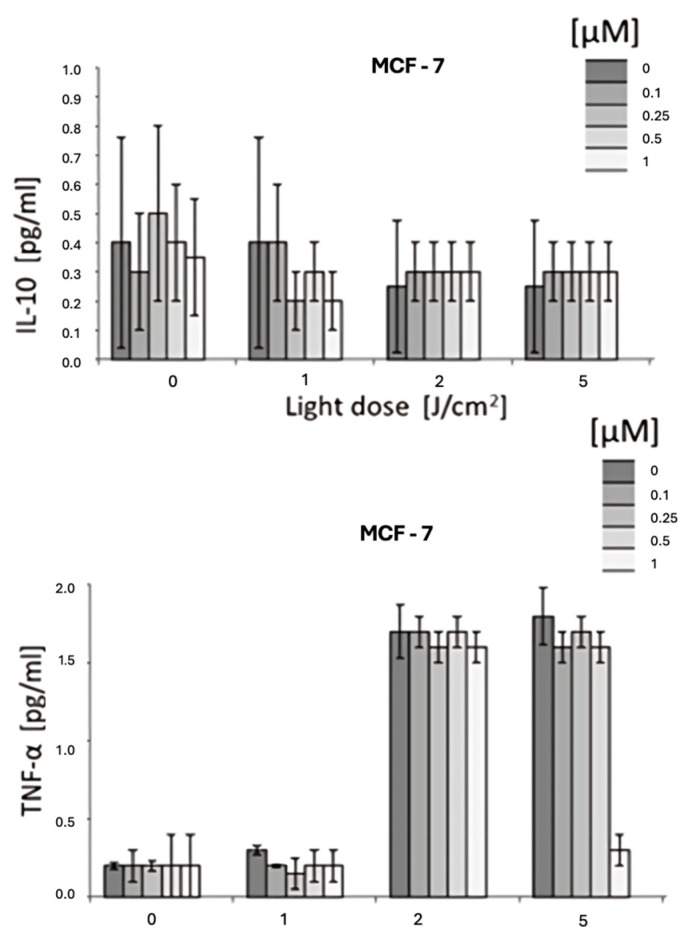
The concentration of IL-10 in the MCF-7 cell line and the concentration of TNF-α in the MCF-7 cell line. The values represent the means ± SD.

- IL-6: A significant reduction in IL-6 levels was observed, with up to a 50% decrease at 1 µM hypericin combined with PDT (*p* < 0.05).
- IL-8: Similarly, IL-8 levels were markedly reduced in response to HYP-PDT at higher light doses and hypericin concentrations.
- IL-10: No statistically significant differences were detected for IL-10 across treatment groups (*p* > 0.05).
- TNF-α: In contrast, TNF-α levels significantly increased following HYP-PDT compared to untreated and dark controls (*p* < 0.05) (Figure 2 and Figure 3).

This is consistent with a shift in the cytokine milieu toward higher TNF-α with lower IL-6 and IL-8, although contributions from reduced viable cell number cannot be excluded.

### 3.4. IC50 Determination for HY-PDT

**Figure 4:**
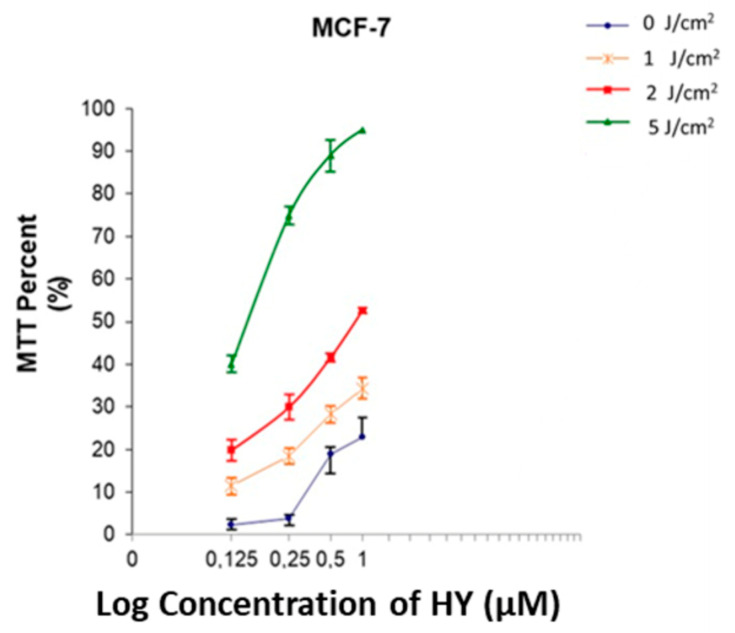
Dose response of MTT signal across hypericin concentrations and light doses. The 50% effect level was not crossed. IC50 not reached within the tested range.

The half-maximal inhibitory concentration (IC50) for hypericin during PDT was calculated using MTT viability data. Within the tested ranges of hypericin and light dose, the response did not cross 50% effect. An IC50 was not reached under these conditions. We therefore report ‘IC50 not reached’ rather than a numerical value (Figure 4). IC50 values based solely on MTT should be interpreted with caution. Incorporation of additional endpoints, such as Trypan Blue at multiple light doses and clonogenic assays for long-term survival, will be necessary to confirm true dose–response relationships.

### 3.5. Cytokine Ratio Analysis

To better understand the balance between pro-inflammatory and anti-inflammatory responses, ratios of IL-6, IL-8, and IL-10 relative to TNF-α were calculated for cells treated with 1 µM hypericin at 5 J/cm^2^ light dose. Table 2 shows the ratios.

The strong increase in TNF-α relative to IL-6 and IL-10 indicates a shift toward a pro-inflammatory environment following HY-PDT.

### 3.6. Cellular Uptake and Localization of Hypericin

**Figure 5:**
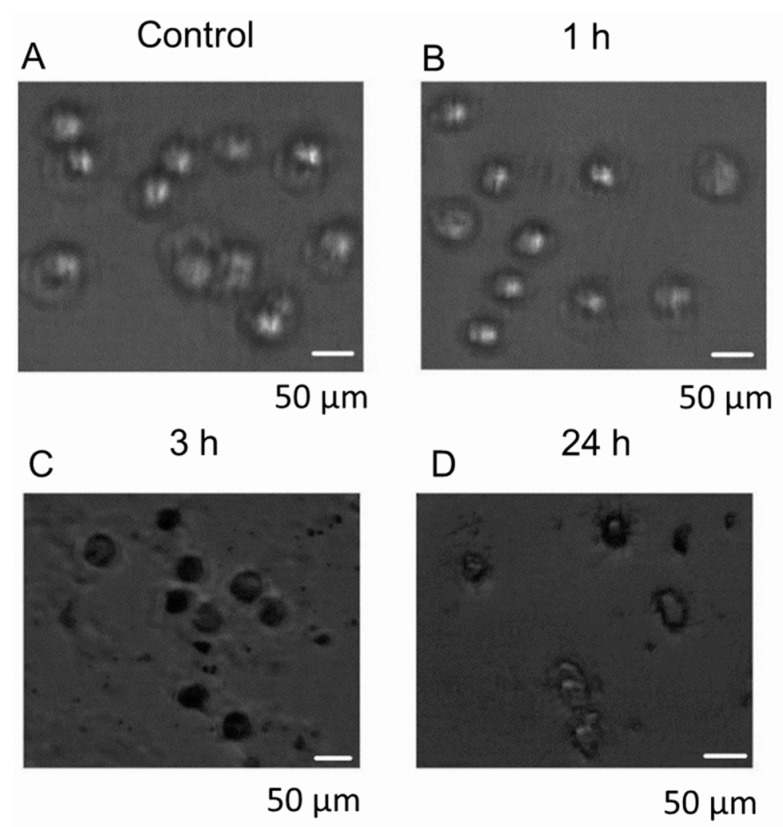
PDT-induced effect of the HY on MCF-7 cells. Light scattering microscopy images show the time-dependent response of the cells to treatment. The dimension of the area displayed is 450 µm × 400 µm. These are not overlay images. Bar scale = 50 µm. (**A**) Control cells before exposure to the device, showing normal morphology. (**B**) Cells after 1 h of exposure, maintaining mostly intact structure with slight surface changes. (**C**) Cells after 3 h of exposure, showing increased darkening and early signs of cell damage. (**D**) Cells after 24 h of exposure, displaying extensive structural degradation and reduced cell density.

**Figure 6:**
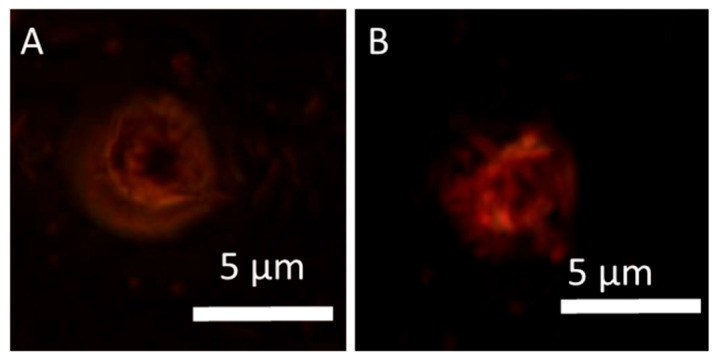
Location of HY into MCF-7 cells after treatment for 3 h: (**A**) fluorescence of HY; (**B**) fluorescence of HY after treatment for 24 h. Magnification 100×. Scale bar = 50 μm. We expect the observed diffraction of light to be the spreading out of waves as they reach and pass through an aperture or around objects. It occurs when the size of the aperture or obstacle is of the same order of magnitude as the wavelength of the incident wave.

**Figure 7:**
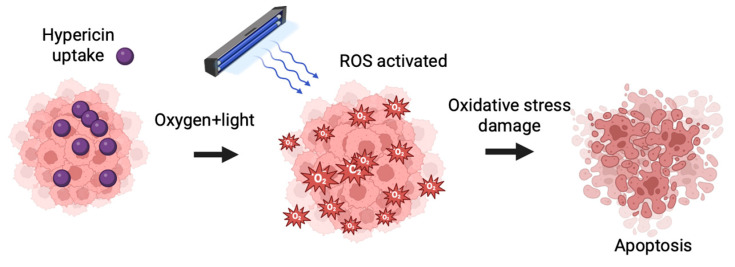
Hypericin photodynamic mechanism.

To visualize hypericin uptake, MCF-7 cells were imaged using fluorescence microscopy. Fluorescence was first detected in the cell membrane and cytoplasm shortly after exposure. Maximum fluorescence intensity was reached within 3 h, indicating efficient uptake and intracellular distribution (Figure 5, Figure 6 and Figure 7). The fluorescence images suggested a homogeneous distribution of hypericin within the cell population, with each captured image covering a surface area of 3.3 × 3.3 mm^2^.

### 3.7. Quantification of Hypericin Uptake

**Figure 8:**
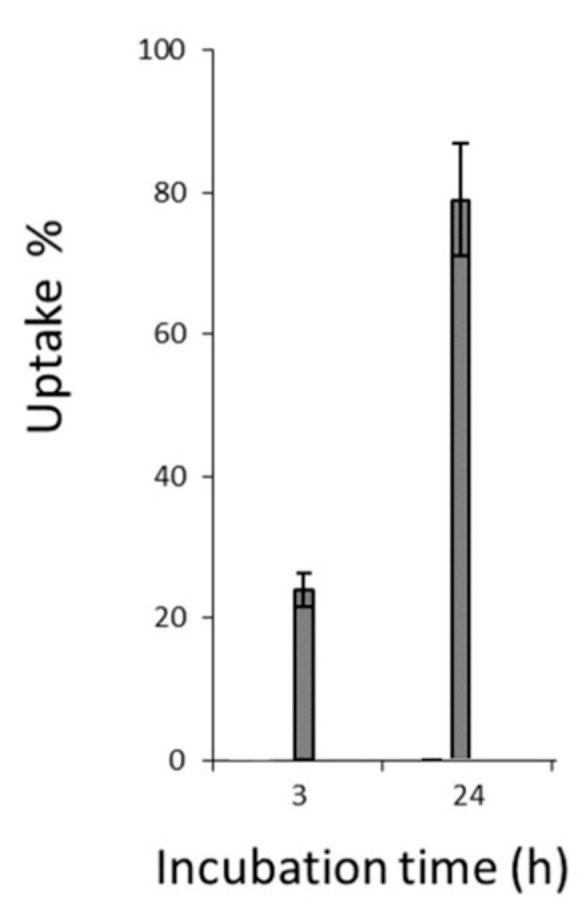
Uptake of Hypericin in the MCF-7 cells in %. The calibration was performed base signal intensity. We consider that the stock solution has a maximum of 100% intensity.

Hypericin uptake by MCF-7 cells was further quantified as a percentage of fluorescence signal intensity, with the stock solution set as 100% reference intensity. Results confirmed progressive uptake over time, plateauing at approximately 3 h post-treatment (Figure 8).

### 3.8. Summary of Findings

1. HYP-PDT decreased cell viability only at higher hypericin concentrations and light doses.
2. IL-6 and IL-8 decreased significantly, while TNF-α increased, indicating a pro-inflammatory shift.
3. Hypericin showed rapid cellular uptake and uniform intracellular distribution.
4. Light diffraction analysis provided insights into how cellular morphology may affect PDT light penetration.

## 4. Discussion

Hypericin (HY) is a naturally occurring antioxidant with strong cytotoxic properties toward various cancer cell lines, including human breast cancer cells [21,22]. Importantly, studies have demonstrated that HYP accumulation is similar in hypoxic and normoxic conditions, such as in A549 and HT-29 cells, indicating that its effectiveness is not diminished in the hypoxic tumor microenvironment [21]. Mirmalek et al. reported that HYP exhibits potent cytotoxic activity in breast cancer (BC) cells, with an IC50 of 5 μg/mL for MCF-7 cells, compared to 20 μg/mL for cisplatin, demonstrating superior efficacy of HYP at lower concentrations [21]. Similarly, treatment with 1–7.5 μM HYP did not alter p53 gene expression in MCF-7 cells, and no changes in Bcl-2 levels were observed in either p53-deficient or wild-type HCT-116 cells after HYP-PDT [22,23,24].

### 4.1. HYP-PDT and Advances in Targeted Cancer Therapy

Modern oncology emphasizes the need for innovative, minimally invasive, and repeatable therapeutic approaches with fewer side effects that do not significantly impair patients’ quality of life. PDT represents one such approach and can be further optimized using nanotechnology to increase tumor specificity. For instance, Mokoena et al. [25] demonstrated that conjugating HYP to gold nanoparticles (AuNPs) significantly enhanced PDT efficacy by promoting cell death primarily through apoptosis [26]. HYP also exerts anti-metastatic and anti-tumor effects even in the absence of light [27,28], while light activation promotes the expression of genes such as ADAMTS9, further suppressing cancer progression [29]. In breast cancer, HYP has been shown to inhibit bone metastases by targeting osteoclast activity via the NFATc1 signaling pathway [30].

The present study demonstrated that HYP-PDT has both phototoxic and immunomodulatory effects on the MCF-7 breast cancer cell line. This is consistent with earlier findings showing that HYP-PDT increases reactive oxygen species (ROS) production, confirming its role as an effective photosensitizer [26,27]. However, the accumulation of HYP in cancer cells may be limited by efflux transporters such as the breast cancer resistance protein (BCRP), which negatively influences HYP uptake and PDT efficacy [31,32,33].

### 4.2. Mitochondrial Targeting and Overcoming Tumor Hypoxia

PDT primarily induces cell death through mitochondrial damage, leading to apoptosis or necrosis [34]. Mitochondria-targeted therapies have demonstrated superior efficacy with reduced systemic toxicity [35]. However, their effectiveness is often compromised by the hypoxic nature of solid tumors, which limits oxygen availability for ROS generation [36,37]. Overcoming hypoxia is therefore a critical challenge for maximizing PDT outcomes. Theodossiou et al. [37] compared two breast cancer cell lines, MCF-7 and MDA-MB-231, and observed that MCF-7 cells were more resistant to HYP-PDT (47 ± 10% viability) compared to MDA-MB-231 cells (27 ± 5%) when treated with 2 µM HYP and irradiated at 530 nm for 45 s at 4 mW/cm^2^ [38]. This highlights the role of genetic and phenotypic differences in treatment response.

### 4.3. Combination Therapies and Clinical Implications

Combining PDT with other therapeutic agents may enhance its effectiveness. Mokoena et al. [38] demonstrated that combining cannabidiol (CBD) with AuNP-HYP-PDT at 5 J/cm^2^ sensitized MCF-7 cells to CBD-induced cytotoxicity [39]. Similarly, other studies showed that HYP-PDT significantly reduced tumor size and increased survival, with greater effects in adenocarcinomas compared to squamous cell carcinomas [40]. PDT can also modulate apoptotic signaling by damaging Bcl-2 and related anti-apoptotic proteins, while activating pro-apoptotic family members [41,42]. This is particularly relevant for triple-negative breast cancer (TNBC), which has limited treatment options. A combination of HYP with Pluronic F127 demonstrated potent cytotoxicity against TNBC cells when treated with 0.4–2.2 µmol/L HYP and 6.3 J/cm^2^ white light [33]. Theodossiou et al. [43] confirmed the phototoxic effects of HYP against both breast and colon adenocarcinoma cells, noting that yellow to orange light (530–550 nm) is optimal for hypericin activation [44,45]. Furthermore, HYP at 10 µg/mL inhibited MCF-7 growth [46] and showed strong selectivity for cancer cells over normal cells, making it a promising candidate for PDT [47,48,49,50,51,52].

### 4.4. Molecular Mechanisms: Apoptosis and Bcl-2 Family Regulation

In our previous research, HYP concentrations of 0.25–0.5 µM significantly decreased hTERT cell viability by up to 78% [40]. HYP-PDT in MCF-7 cells has been associated with downregulation of Bcl-xl and upregulation of Bax, though no effect was observed on Bcl-2 levels in HCT-116 cells with different p53 statuses [48]. These findings suggest that anti-apoptotic Bcl-2 family members play a complex role in PDT-induced apoptosis. Studies by He et al. [48] revealed that parental cells exhibited high apoptosis rates post-PDT, whereas Bcl-2-transfected cells showed significantly reduced apoptosis, as measured by DNA fragmentation and flow cytometry. Kim et al. [49] further demonstrated that Bcl-2 transfection increased both Bcl-2 and Bax expression. Photochemical mitochondrial damage elevated the Bax:Bcl-2 ratio, a critical determinant of apoptosis initiation [50]. Srivastava et al. [51] confirmed that Bcl-2 overexpression enhanced PDT-mediated caspase activation and apoptosis, highlighting Bcl-2 as a key regulatory target in PDT [52].

### 4.5. Natural Compounds and Future Nanomedicine Applications

Natural products such as HYP hold promise for increasing drug uptake and improving chemotherapy efficacy [52,53]. The development of self-targeting nanomedicine platforms may enhance PDT selectivity and reduce systemic toxicity [54]. Such approaches could revolutionize clinical applications, especially for aggressive breast cancers.

### 4.6. Cytokine Modulation and Immunological Implications

Cytokines play a critical role in breast cancer biology and immune responses to therapy. Our study observed a decrease in IL-6 and IL-8 levels and an increase in TNF-α following HY-PDT, indicating a shift toward a pro-inflammatory immune environment. Previous studies have reported significant differences in serum IL-6 levels between healthy donors and early-stage breast cancer patients [55,56]. However, IL-6 exhibits complex and sometimes contradictory effects on breast cancer cells. Badache and Hynes found that IL-6 inhibited T47D cell proliferation via STAT3 activation [57]. Danforth and Sgagias reported additive growth inhibition in MCF-7 cells with IL-6 treatment [58]. Conversely, IL-6 did not affect MCF-7 proliferation directly but enhanced estrone sulfate-induced growth [59,60]. Conze et al. showed that IL-6 promotes multidrug resistance in MCF-7 cells [59]. The molecular mechanism of IL-6 in breast cancer is likely context-dependent, involving interactions between the STAT3-dependent pathway and the growth-promoting MAPK/PI3K pathway [61,62,63,64,65]. IL-8, another key cytokine, remains a controversial biomarker. De Sanctis et al. found no significant changes in IL-8 levels after four weeks of radiotherapy [65], whereas Muraro et al. reported a transient increase following stereotactic body radiotherapy [66]. Similarly, IL-10 levels in breast cancer patients have produced conflicting findings across multiple studies [67,68,69,70]. These discrepancies highlight the complexity of cytokine dynamics in the tumor microenvironment and underscore the need for further research [71]. Our findings suggest that HYP-PDT modulates cytokines in a way that could potentially enhance anti-tumor immune responses. However, the exact clinical implications require further investigation. Observed decreases in IL-6 and IL-8 may arise from reduced viable cell mass or metabolic arrest. Future experiments will report secretion rates normalized to viable cell counts or total protein, include LDH release to quantify lysis, and profile additional mediators of immunogenic cell death.

### 4.7. Immunogenic Cell Death and STAT3/NF-κB Pathway Modulation by HYP-PDT

Our results are compatible with, but do not establish, immunogenic cell death [19,28,40]. These signals are known to recruit and activate dendritic cells, leading to effective priming of cytotoxic CD8+ T cells and enhanced adaptive immune responses. The cytokine profile observed in this study, characterized by reduced IL-6 and IL-8 levels with increased TNF-α secretion, suggests that HYP-PDT shifts the tumor microenvironment from a STAT3-dependent pro-tumor state toward an NF-κB–driven pro-inflammatory response. STAT3 activation has been implicated in tumor growth, immune evasion, and suppression of dendritic cell maturation, while NF-κB signaling is essential for T cell activation and antitumor immunity [61,62,63,64]. By downregulating IL-6, a key activator of the STAT3 pathway, HYP-PDT may disrupt these immunosuppressive circuits, while elevated TNF-α promotes NF-κB–mediated inflammatory signaling that favors immune cell recruitment and activation. These dual effects suggest that HYP-PDT not only induces direct cytotoxicity but also reprograms cytokine networks to enhance the immunogenicity of breast cancer cells. Future studies incorporating dendritic cell and T cell co-culture systems will be essential to confirm whether these molecular changes translate into functional T cell activation. Such mechanistic insights could provide a rationale for combining HYP-PDT with immune checkpoint inhibitors or STAT3-targeted therapies to achieve synergistic antitumor effects [19,39,61,62,63,64]. As this is a preliminary study conducted in a monoculture system, future research will incorporate dendritic cell and T cell co-culture models to directly evaluate antigen presentation and T cell activation, as recommended by the reviewer, thereby providing a more comprehensive understanding of the immunomodulatory potential of HY-PDT. Such mechanistic insights could provide a rationale for combining HY-PDT with immune checkpoint inhibitors or STAT3-targeted therapies to achieve synergistic antitumor effects [19,39,61,62,63,64]. Planned assays include calreticulin exposure, ATP and HMGB1 release, and cytokine normalization to viable cell counts, followed by dendritic cell co-culture to test functional antigen presentation.

### 4.8. Study Significance and Future Directions

This study is the first to demonstrate both the cytotoxic and immunomodulatory effects of HYP-PDT on the MCF-7 breast cancer cell line. The results indicate that treatment outcomes depend on both hypericin concentration and light dose, emphasizing the importance of precise treatment parameter optimization. Future experiments will extend the dose range or adjust light parameters to capture the full sigmoidal response needed for robust IC metrics, or will report alternative parameters such as EC25 or area-under-the-curve. This study was limited to MCF-7 cells, which represent ER+ luminal breast cancer. Future work should extend to additional models, such as triple-negative MDA-MB-231 cells and normal mammary epithelial cells, to assess whether observed effects are generalizable or subtype-specific.

Given the dual role of PDT in directly killing cancer cells and modulating the immune response, future studies should focus on:

1. Validating these findings in in vivo breast cancer models.
2. Exploring combination therapies with chemotherapy, immunotherapy, or targeted drugs.
3. Investigating cytokine regulation as a biomarker for treatment response.

Our results suggest that Hypericin PDT may be further optimized through nanotechnology-based delivery platforms and multimodal therapies. For example, Hypericin PDT could synergistically enhance efficiency in nanodrugs designed for hyperthermia applications in breast cancer [72]. Exploring such synergies in preclinical models may accelerate translation toward clinical protocols. The cytokine panel in this study was limited to IL-6, IL-8, IL-10, and TNF-α. Expanding to additional mediators such as IFN-γ, TGF-β, GM-CSF, or broad multiplex proteomics would provide a more comprehensive picture of the immunomodulatory effects. Linking these shifts to STAT3 versus NF-κB signaling pathways will help clarify whether Hypericin PDT favors immune activation or suppression. The study has several limitations: (i) it was conducted in a monoculture model without immune components; (ii) no in vivo validation was performed; (iii) cytokine secretion was measured without normalization to secretory capacity per cell; (iv) drug efflux pumps such as BCRP may have interfered with hypericin uptake. These factors should be addressed in follow-up studies to strengthen translational relevance. While these in vitro findings are promising, translation to the clinic requires caution. Standardized dosimetry, patient-relevant models such as 3D spheroids or organoids, and validation in animal models are needed to evaluate therapeutic potential. Future combination strategies with chemotherapy, immune checkpoint inhibitors, or STAT3-targeted drugs may enhance efficacy. Only after such preclinical work can clinical trials be considered.

## 5. Conclusions

This study demonstrates, for the first time, that hypericin-mediated photodynamic therapy (HY-PDT) exerts both cytotoxic and immunomodulatory effects on MCF-7 human breast adenocarcinoma cells. HYP-PDT effectively reduced cell viability in a concentration- and light dose-dependent manner. Importantly, HYP-PDT modulated cytokine secretion, significantly decreasing IL-6 and IL-8 levels while increasing TNF-α, suggesting a shift toward a pro-inflammatory microenvironment that could enhance anti-tumor immune responses. These findings highlight the potential of HYP-PDT as a dual-action therapeutic strategy, combining direct tumor cell destruction with modulation of immune signaling pathways. Given the complex role of cytokines in breast cancer progression, these results provide a foundation for further exploration of HYP-PDT as an adjunct to conventional treatments such as chemotherapy, radiotherapy, or immunotherapy. Future studies should include in vivo models and clinical trials to validate these results, optimize treatment parameters, and fully evaluate the translational potential of HYP-PDT in breast cancer therapy. With additional research, HYP-PDT could emerge as a minimally invasive, targeted treatment option with fewer side effects and improved patient outcomes.

## Figures and Tables

**Table 1:** Viability (%) of MCF-7 breast cancer cells treated with hypericin in the dark (0 J/cm^2^).

Hypericin (µM)	Trypan Blue Viability (%)	MTT Metabolic Activity (%)
0 (control)	98.9 ± 0.3	95 ± 3
0.125	95 ± 2.3	98 ± 5
0.25	93 ± 2	93 ± 4
0.5	84 ± 15	86 ± 6
1.0	87 ± 5	84 ± 2

**Table 2:** Ratios of cytokine concentrations after HYP-PDT treatment.

Ratio	Value
IL-6:TNF-α	0.1:1.7
IL-8:TNF-α	3.6:1.7
IL-10:TNF-α	0.1:1.7

## Data Availability

All new data created in this study are available in this manuscript.

## Abbreviations

The following abbreviations are used in this manuscript:

Abbreviation	Full form
BC	Breast cancer
PDT	Photodynamic therapy
HYP	Hypericin
HYP-PDT	Hypericin-mediated photodynamic therapy
PS	Photosensitizer
^1^O_2_	Singlet oxygen
ROS	Reactive oxygen species
H_2_O_2_	Hydrogen peroxide
OH·	Hydroxyl radical
O_2_·^−^	Superoxide anion
MCF-7	Human breast adenocarcinoma cell line MCF-7
IL	Interleukin
IL-1	Interleukin 1
IL-2	Interleukin 2
IL-6	Interleukin 6
IL-8	Interleukin 8
IL-10	Interleukin 10
IL-11	Interleukin 11
IL-12	Interleukin 12
IL-17A	Interleukin 17A
IL-18	Interleukin 18
IL-19	Interleukin 19
IL-20	Interleukin 20
IL-22	Interleukin 22
IL-23	Interleukin 23
IFN-γ	Interferon gamma
TGF-β	Transforming growth factor beta
IRF-1	Interferon regulatory factor 1
IRF-2	Interferon regulatory factor 2
gp130 or GP130	Glycoprotein 130
MMP-1	Matrix metalloproteinase 1
TNF-α	Tumor necrosis factor alpha
DAMPs	Danger-associated molecular patterns
IC50	Half-maximal inhibitory concentration
ATCC	American Type Culture Collection
DMEM/F12	Dulbecco’s Modified Eagle’s Medium: F-12
EMEM	Eagle’s Minimum Essential Medium
FBS	Fetal bovine serum
EDTA	Ethylenediaminetetraacetic acid
PBS	Phosphate-buffered saline
DMSO	Dimethyl sulfoxide
IR	Infrared
CO_2_	Carbon dioxide
OD	Optical density
SD	Standard deviation
ANOVA	Analysis of variance
TNBC	Triple negative breast cancer
AuNPs	Gold nanoparticles
BCRP	Breast cancer resistance protein
MRP1	Multidrug resistance-associated protein 1
NFATc1	Nuclear factor of activated T cells c1
MAPK	Mitogen-activated protein kinase
PI3K	Phosphoinositide 3-kinase
STAT3	Signal transducer and activator of transcription 3
ADAMTS9	A disintegrin and metalloproteinase with thrombospondin motifs 9
HPPH	2-(1-hexyloxyethyl)-2-devinyl pyropheophorbide a
SnEt2	Tin ethyl etiopurpurin
LuTex	Lutetium texaphyrin
ISO	International Organization for Standardization
CBD	Cannabidiol
